# DeSUMOylation Controls Insulin Exocytosis in Response to Metabolic Signals

**DOI:** 10.3390/biom2020269

**Published:** 2012-05-24

**Authors:** Elisa Vergari, Gregory Plummer, Xiaoqing Dai, Patrick E. MacDonald

**Affiliations:** 1 BIOTEC TU-Dresden, Tatzberg 47/49 01307, Germany; Email: elisa.vergari@ocdem.ox.ac.uk (E.V.); 2 Alberta Diabetes Institute and Department of Pharmacology, University of Alberta, Edmonton T6G 2E1, AB, Canada; Email: gplummer@ualberta.ca (G.P.); xdai@ualberta.ca (X.Q.D.)

**Keywords:** insulin, exocytosis, SUMOylation, SENP1, NADPH, redox

## Abstract

The secretion of insulin by pancreatic islet β-cells plays a pivotal role in glucose homeostasis and diabetes. Recent work suggests an important role for SUMOylation in the control of insulin secretion from β-cells. In this paper we discuss mechanisms whereby (de)SUMOylation may control insulin release by modulating β-cell function at one or more key points; and particularly through the acute and reversible regulation of the exocytotic machinery. Furthermore, we postulate that the SUMO-specific protease SENP1 is an important mediator of insulin exocytosis in response to NADPH, a metabolic secretory signal and major determinant of β-cell redox state. Dialysis of mouse β-cells with NADPH efficiently amplifies β-cell exocytosis even when extracellular glucose is low; an effect that is lost upon knockdown of SENP1. Conversely, over-expression of SENP1 itself augments β-cell exocytosis in a redox-dependent manner. Taken together, we suggest that (de)SUMOylation represents an important mechanism that acutely regulates insulin secretion and that SENP1 can act as an amplifier of insulin exocytosis.

## 1. Introduction

**Figure 1:**
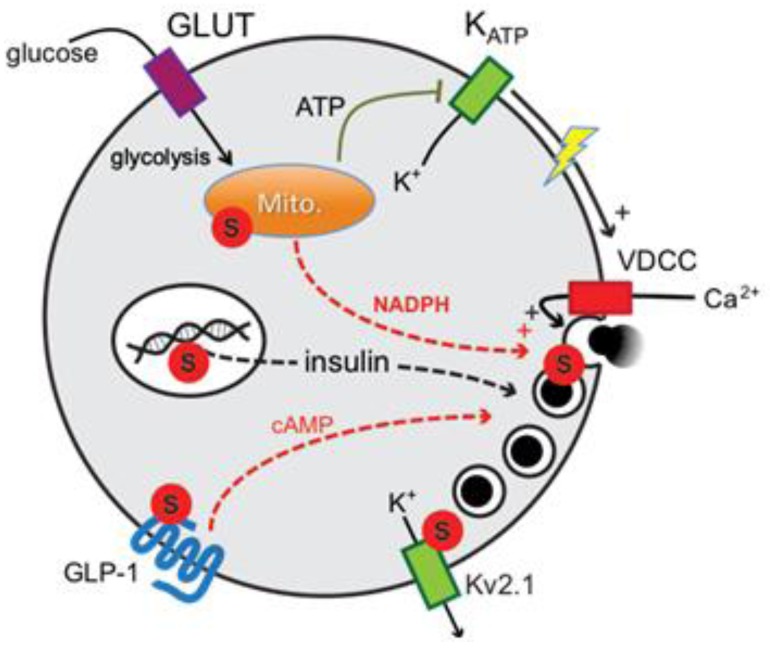
Potential sites of regulation by SUMOylation in the mechanism of glucose-stimulated insulin secretion from pancreatic β-cells. The general mechanism regulating insulin secretion through triggering and amplifying pathways is shown. SUMOylation (**S**) may control insulin gene transcription, mitochondrial (Mito.) function, GLP-1 receptor signaling/localization, Kv2.1 channel function and downstream exocytosis in response to metabolic signals.

Insulin secretion from pancreatic islet β-cells is tightly regulated by many factors including nutrients, hormones, and neurotransmitters. Increased insulin release in response to glucose is central to energy homeostasis, and is impaired in diabetes [1]. At least two pathways control glucose-stimulated insulin secretion in β-cells and are usually referred to as ‘*triggering*’ and ‘*amplifying*’ pathways [2,3,4] (Figure 1). The triggering pathway is characterized by events leading to an increase in intracellular Ca^2+^ following the metabolic generation of ATP, inhibition of ATP-sensitive K^+^ (K_ATP_) channels, and subsequent activation of voltage-dependent Ca^2+^ channels (VDCCs) [5]. The secretory response to Ca^2+^ can be amplified by several receptor-mediated and metabolic signals [2,3,4]. Key among these is the generation of cAMP through the action of incretin hormones such as glucagon-like peptide-1 (GLP-1) [6,7,8], while several candidate metabolic signals have been proposed [9,10]. Among these, NADPH derived from the mitochondrial export of malate and citrate is perhaps the strongest candidate [9,10,11,12]. The downstream mechanism by which NADPH affects insulin secretion is unclear, but may involve a glutaredoxin-dependent pathway [13,14] which presumably transduces the generation of these reducing equivalents into an action on the secretory machinery through the cycling of reduced/oxidized glutathione couples [15].

SUMOylation, the post-translational modification of a target protein by the covalent attachment of SUMO (small ubiquitin like modifier), modulates target activity, protein-protein interactions, and sub-cellular localization [16,17]. Much work has been devoted to understanding the nuclear effects of SUMOylation, and indeed in pancreatic β-cells SUMOylation of the transcription factors Pdx-1 and MafA regulates their localization and control of insulin gene transcription [18,19]. Recently, extra-nuclear roles have been demonstrated, including the regulation of synaptic transmission [20,21], ion channel activity [22,23,24,25], and mitochondrial function and morphology [26,27,28]. Thus, the SUMOylation pathway is emerging as a regulatory mechanism that can control many key cellular functions.

SUMOylation is readily reversible through the action of the sentrin-specific SUMO proteases (SENPs), of which there are at least six identified in mammalian cells (SENP1-3, 5-7) [29]. While the mechanisms regulating SENP activity remain unclear [29,30], recent reports suggest that the enzyme is regulated by the intracellular redox state through the formation of di-sulfide bonds between SENP1 monomers (at least two cysteine residues are implicated, C603 and C613) [31]. This is suggested to modulate the cellular response to reactive oxygen species [32], and could be particularly relevant to pancreatic β-cell function since redox changes are implicated both in the normal physiology of insulin secretion and in diabetes pathophysiology [33,34]. Here we discuss the effects of SUMO1, and the role of (de)SUMOylation (particularly through SENP1), in the control of insulin secretion. Furthermore, we present evidence that SENP1 is an important mediator of insulin exocytosis in response to the key metabolic signal, NADPH.

## 2. Results and Discussion

### 2.1. SUMO1 and Insulin Secretion

#### 2.1.1. Acute and Reversible Inhibition of Insulin Exocytosis by SUMOylation

**Figure 2:**
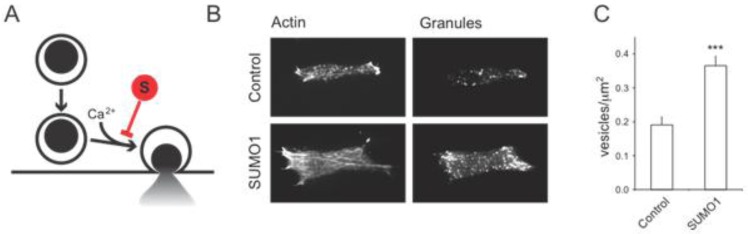
SUMOylation inhibits the exocytosis of secretory granules, without affecting granule trafficking to the plasma membrane. (**A**) SUMOylation (**S**) acts downstream of insulin granule targeting to the plasma membrane to inhibit Ca^2+^-dependent exocytosis. Up-regulating SUMO1 blocks downstream exocytosis and results in a build-up of secretory granules at the plasma membrane. This can be seen by total-internal reflection fluorescence (TIRF) imaging of cortical actin and secretory granules labeled with NPY-mCherry within ~100 nm of the plasma membrane. Representative images are shown in (**B**), and quantified data is shown in (**C**). *** p < 0.001 compared with control.

Up-regulation of SUMOylation in pancreatic islets or β-cells inhibits insulin secretion stimulated by glucose [35] or activation of the GLP-1 receptor [36]. This occurs in the absence of changes in VDCC activity and intracellular Ca^2+^ responses, suggesting that much of the upstream mechanism (*i.e.*, glucose metabolism, electrical activity and Ca^2+^ entry) controlling insulin secretion remains intact [35]. In order to be secreted, insulin granules must first be trafficked to the plasma membrane [37,38]. This process as well is intact following SUMO1 over-expression in insulin-secreting cells. In fact, cells over-expressing SUMO1 have more secretory granules localized to the plasma membrane (Figure 2). Thus, while the insulin granule trafficking and glucose-dependent Ca^2+^ responses are intact, insulin release is blunted following SUMO1 over-expression [35,36]. This would suggest that SUMO1 acts far downstream in the secretory pathway, likely by inhibiting the exocytosis of insulin granules in response to the intracellular Ca^2+^ signal [35]. As such, the increased secretory granule density at the plasma membrane of insulin secreting cells (INS-1 insulinoma cells are shown in Figure 2) is a ‘traffic jam’ resulting from efficient plasma membrane targeting of insulin granules which are then unable to undergo exocytosis and release their cargo.

Since it is possible that the up-regulation of SUMO1 expression could inhibit insulin secretion by down-regulating the expression of insulin itself [18,19], it is important to note that we observed no change in insulin content upon over-expression of SUMO for 24–48 hours. While a more chronic over-expression of SUMO1 may result in decreased insulin expression, and indeed was observed in insulinoma cells stably over-expressing GFP-SUMO1 [36]. The SUMO1-dependent inhibition of insulin exocytosis certainly occurs acutely however, and is likewise rapidly reversible, as the direct intracellular dialysis of recombinant SUMO1 into β-cells blocks exocytosis within 1–2 minutes and this can be reversed (with a similar time course) by the direct infusion of SENP1 [35]. These findings are important for two reasons: (1) the regulation of exocytosis in these cells is dependent on SUMO-conjugation to a target (*i.e.*, is reversible by SUMO cleavage); and (2) the effects of SUMOylation on exocytosis are very rapid, precluding a role for changes in gene expression *per se*.

#### 2.1.2. Possible SUMO Targets Inhibiting Insulin Secretion

There are several points at which SUMOylation may impact insulin secretion (Figure 1). SUMOylation can control nuclear signaling in β-cells [39], including regulation of the key insulin gene transcription factors MafA and Pdx1 [18,19]. Somewhat further ‘downstream’, SUMOylation has been demonstrated to control mitochondrial fission and function [26,28]. Although unexplored in the context of insulin secretion and β-cell function, this would be expected to modulate the generation of metabolic signals that are essential for insulin secretion. While our recent findings suggest that mitochondrial function may be more-or-less intact following SUMO1 over-expression given normal islet Ca^2+^ responses [35], a more detailed analysis of mitochondrial activity and morphology would be required to support a lack of effect of SUMO1 on β-cell mitochondria.

One recent report demonstrated the SUMOylation-dependent trafficking of the G-protein-coupled GLP-1 receptor [36]. A key factor in promoting postprandial insulin secretion, GLP-1 is secreted from intestinal L-cells following a meal and acts to augment the β-cell secretory response to circulating glucose primarily through the generation of cAMP (reviewed extensively [6,7,40]). Rajan *et al*. [36] showed that SUMOylation of the GLP-1 receptor prevents its cell surface trafficking, resulting in impaired cAMP generation and insulin secretion in response to GLP-1. Intriguingly, these authors also demonstrated the up-regulation of SUMO mRNA (and that of the SUMO-conjugating enzyme Ubc9) following exposure of mouse islets to high glucose, raising the possibility that increased SUMOylation could contribute to the reduced insulin secretion observed in diabetes.

**Figure 3:**
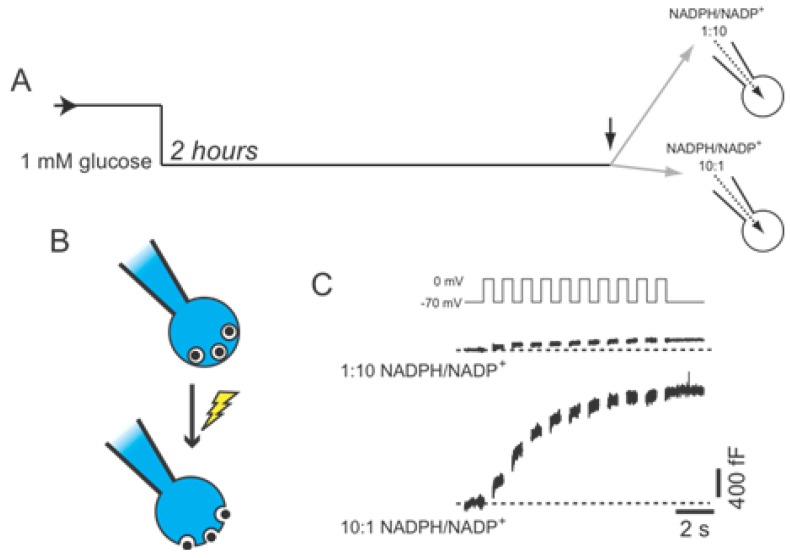
Amplification of β-cell exocytosis by NADPH. (**A**) Mouse β-cells were maintained at a non-stimulatory glucose concentration (1 mM) for 2 hours prior to intracellular dialysis of NADPH/NADP^+^ (at molar ratios of either 1:10 or 10:1) and whole-cell patch-clamp measurement of exocytosis; (**B**) Exocytosis is measured as the increase of cell surface area (capacitance) that occurs following membrane depolarization, opening of voltage-dependent Ca^2+^ channels, and subsequent fusion of secretory granules with the plasma membrane; (**C**) The exocytotic response to a series of ten membrane depolarizations remained low under the reduced NADPH condition (1:10), but is significantly amplified by elevation of NADPH (10:1) even when glucose remains low (1 mM).

**Figure 4:**
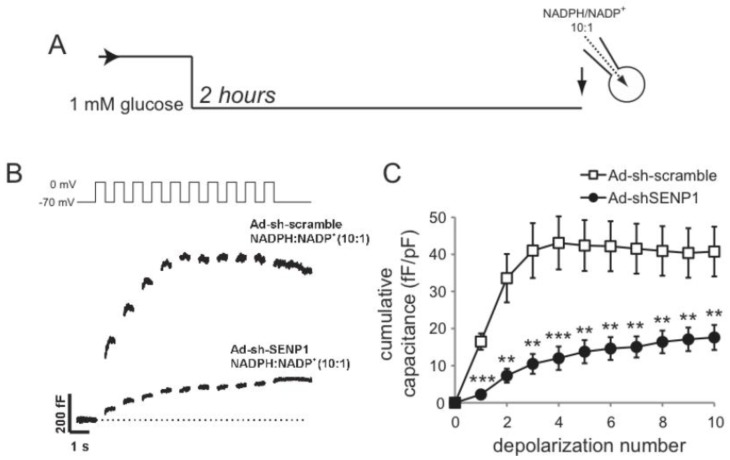
SENP1 is required for NADPH-dependent amplification of exocytosis. (**A**) Mouse β-cells were maintained at a non-stimulatory glucose concentration (1 mM) for 2 hours prior to intracellular dialysis of NADPH/NADP^+^ at a molar ratio 10:1 and whole-cell patch-clamp measurement of exocytosis; (**B**,**C**) Representative traces (**B**) of exocytotic responses and averaged data (**C**) in control cells (Ad-sh-scramble) or following SENP1 knockdown (Ad-sh-SENP1). ** p < 0.01 and *** p < 0.001 compared with Ad-sh-scramble.

**Figure 5:**
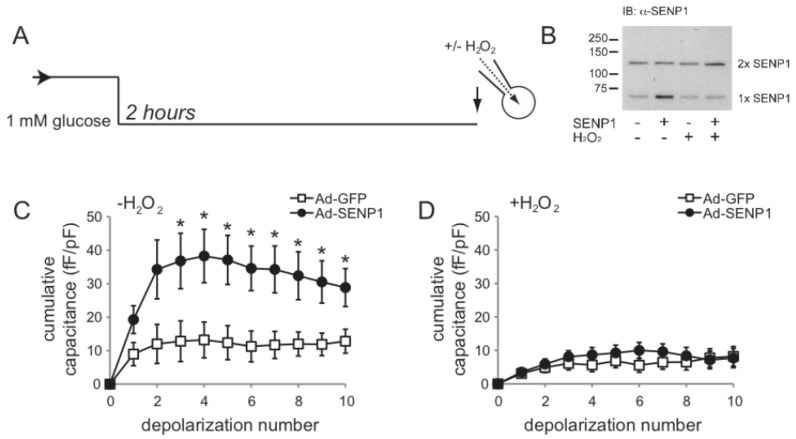
SENP1 amplification of β-cells is redox-dependent. (**A**) Mouse β-cells, infected with adenovirus expressing GFP (Ad-GFP) or SENP1 (Ad-SENP1, which co-expresses GFP), were maintained in non-stimulatory glucose (1 mM) for 2 hours prior to whole-cell patch-clamp. In some experiments, H_2_O_2_ (200 µM) was infused directly into cells at the time of the experiment; (**B**) Immunoblotting of protein lysates from INS-1 832/13 cells demonstrates the up-regulation of monomeric SENP1 (with Ad-SENP1) and its subsequent dimerization in the presence of H_2_O_2_ (200 µM); (**C**,**D**) Over-expression of SENP1 recapitulates the effect of NADPH to amplify β-cell exocytosis (**C**), an effect that is lost under oxidizing conditions (**D**). * p < 0.05 compared with Ad-GFP.

Given that SUMO1 can inhibit insulin exocytosis downstream of the sites mentioned above, we have focused our attention on potential SUMOylation targets at the exocytotic site. Our initial work focused on the voltage-dependent K^+^ (Kv) channel Kv2.1, which is highly expressed in rodent and human β-cells and mediates action potential repolarization [41,42,43,44], since related K^+^ channels are regulated by SUMOylation [22,23]. Indeed, both cloned Kv2.1 and the native channel in rodents and humans is inhibited by SUMOylation [24] of a C-terminal lysine (K470) [25]. While inhibition of Kv2.1 currents in itself cannot account for the ability of SUMO1 to block exocytosis, since our exocytosis measurements (such as those in Figure 3, Figure 4, Figure 5) are carried out under conditions where the cell membrane potential is ‘clamped’, it is interesting to note that the Kv2.1 channel can regulate β-cell exocytosis independent of its electrical function, through a direct interaction with the exocytotic protein syntaxin 1A [45,46]. It will thus be interesting to determine whether SUMOylation of Kv2.1 alters its role in β-cell exocytosis and its interaction with syntaxin 1A, which we have also recently identified to itself be SUMOylated in pancreatic islets (unpublished). Finally, as discussed in further detail below, our original screen for exocytotic proteins that interact with SUMO1 in insulin-secreting cells identified a likely candidate to be synaptotagmin VII [35], which is the primary Ca^2+^-sensor for insulin exocytosis [47] and possesses two candidate SUMOylation sites; one each in close proximity to its two Ca^2+^-binding domains.

#### 2.1.3. DeSUMOylation Is Required for Insulin Secretion

The cellular and molecular pathways that modulate SUMOylation at the exocytotic site are unknown, but our work suggests that SUMOylation acts as a ‘brake’ on exocytosis, perhaps to prevent unwanted insulin secretion when circulating glucose levels are low. The implication of this is interesting, as it suggests that the acute facilitation of exocyosis is regulated by *removal* of SUMO-conjugates (*i.e.*, deSUMOylation). Indeed, synaptotagmin VII appears to be basally SUMOylated at non-stimulatory glucose concentrations [35]. Following glucose stimulation, we observed a transient deSUMOylation of synaptotagmin VII (which could be replicated by SENP1 over-expression). Indeed, deSUMOylation is required for the glucose-dependent amplification of insulin exocytosis since block of deSUMOylation (by either up-regulating the conjugating enzyme Ubc9, or knockdown of SENP1) prevents glucose-dependent amplification of β-cell exocytosis. Furthermore, up-regulation of SENP1 is itself able to amplify the β-cell exocytotic response to Ca^2+^, recapitulating the effect of glucose-stimulation (seen also in Figure 5). Thus, the deSUMOylating enzyme is both required and sufficient in glucose-dependent amplification of β-cell exocytosis, although the molecular and metabolic pathways linking glucose-stimulation and deSUMOylation are unknown.

### 2.2. SENP1 Is Required for NADPH-Dependent Insulin Exocytosis

One possible link between glucose-stimulation and the enhancement of insulin exocytosis is NADPH, which can directly augment β-cell exocytotic responses [13,14]. We show here that NADPH is able to enhance the exocytotic response of β-cells even in the absence of stimulatory glucose (Figure 3). In these cells NADPH is dialysed directly into mouse β-cells (at a molar ratio of ether 1:10 or 10:1 with NADP^+^) through a patch-clamp pipette, following which the cell is subjected to series of membrane depolarizations to activate Ca^2+^ channels, allowing Ca^2+^ influx and the stimulation of exocytosis [48]. In this case, exocytosis is monitored as increased cell surface area (called capacitance) upon the fusion of secretory granules.

To determine whether SENP1 is required for the NADPH-dependent amplification of insulin exocytosis a knock-down approach was used [35]. NADPH-dependent amplification of exocytosis was observed in β-cells infected with a control adenovirus (Ad-sh-Scramble; n = 28) (Figure 4). Using a SENP1-targed shRNA adenovirus (Ad-sh-SENP1) described previously [35], we find that NADPH fails to enhance exocytosis following SENP1 knock-down, where the response was impaired by 57% compared to the control (n = 21, p < 0.01) (Figure 4). Conversely, SENP1 up-regulation augments exocytosis in mouse β-cells in the absence of a glucose-stimulus or NADPH (n = 12) compared with that seen upon expression of GFP only (n = 8, p < 0.05) (Figure 5). We saw no difference in VDCC activity following either SENP1 knockdown or over-expression (not shown). Since intracellular redox state is suggested to regulate SENP1 activity, possibly by controlling SENP1 dimerization [31,32], we examined whether SENP1 amplification of exocytosis was dependent upon intracellular redox. For this, hydrogen peroxide was used, which we show to promote SENP1 dimerization in INS-1 832/13 insulinoma cells (Figure 5B). When 200 µM of H_2_O_2_ were added in the pipette solution SENP1 over-expression was no longer able to amplify the exocytotic response of mouse β-cells (n = 13) compare to the GFP control (n = 11) (Figure 5).

## 3. Experimental Section

### 3.1. Recombinant Adenoviruses, Islet Isolation and Cell Culture

Our recombinant adenoviruses expressing either SENP1 (Ad-SENP1) or a SENP1-targeted shRNA (Ad-sh-SENP1) were described and characterized previously [35]. These co-express GFP to allow identification of infected cells. Adenoviruses expressing GFP alone (Ad-GFP) or a scrambled shRNA sequence (Ad-sh-Scrambled) served as controls.

Pancreatic islets were isolated from male C57/BL6 mice by collagenase (1 mg/mL) digestion and handpicked as previously [35,50]. Islets were then dispersed to single cells by shaking 11 min in Ca^2+^-free media (138 mM NaCl, 5.6 mM KCl, 1.2 mM MgCl_2_, 5 mM HEPES, 3 mM glucose, 1 mM EGTA, 1 mg/mL albumin). Cells were plated into 35 mm culture dishes in RPMI 1640 with L-glutamine, 10% FBS, and 100 units/mL penicillin/streptomycin. When primary cells and cell lines were infected with Ad-GFP or Ad-SENP1 they were cultured 2 days prior to experiments while when infected with Ad-sh-Scrambled or Ad-sh-SENP1 they were cultured for 3 days to allow for SENP1 knockdown. All animal experiments were approved by the animal care and use committee at the University of Alberta.

INS1 832/13 insulinoma cells (a gift from Prof. C. Newgard, Duke University) were maintained in RPMI1640 medium with 11.1 mM glucose and 2 mM L-glutamine, supplemented with 10% FBS, 10 mM HEPES, 100 U/mL penicillin/streptomycin, 1 mM sodium pyruvate, 50 μM β-mercaptoethanol, in a humidified atmosphere (5% CO_2_, 37 °C).

### 3.2. Western Blotting

INS1 832/13 cells infected with Ad-GFP or Ad-SENP1 were incubated in KRBH buffer (135 mMNaCl, 3.6 mMKCl, 0.5 mM MgCl_2_, 0.5 mM NaH_2_PO_4_, 10 mM HEPES, 2 mM NaHCO_3_, 1.5 mM CaCl_2_, 0.1% BSA, 11.1 mM glucose, pH = 7.4) with or without 1 mM hydrogen peroxide (H_2_O_2_, Sigma) for 30 min in the dark at 37 °C. Cells were harvested and lysed in Cell-Lytic-M buffer (Sigma-Aldrich), supplemented with DTT and protease inhibitor cocktail (Bio Basic Inc.) and kept on ice for 30 min. Lysates were separated in 8% SDS-PAGE and transferred to a polyvinylidenedifluoride (PVDF) membrane (Millipore, Billerica, MA) followed by blocking with 5.0% nonfat dry milk in TBST (150 mM NaCl, 50 mM Tris, and 0.1% Tween 20, pH = 7.4) for 1 h at room temperature. The primary antibodies were a mouse monoclonal anti-SENP1 (C12) (1:700, Santacruz) and mouse monoclonal anti-β-tubulin (1:40,000, Sigma; not shown) at 4 °C overnight. After washing with TBST, the membrane was incubated with peroxidase-linked anti-mouse IgG (Amersham Bioscience; 1:5,000) for 1h at room temperature. Detection was done by an enzymatic chemiluminescence (ECL) kit (Amersham Bioscience) and exposure to X-ray film (Fujifilm, Tokyo, Japan).

### 3.3. Electrophysiology

Changes in membrane capacitance were monitored in whole-cell configuration using EPC10 patch-clamp amplifier controlled with PatchMaster software (HEKA Electronik, Lambrecht, Germany). Experiments were performed at 32–35 °C. Extracellular solution contained (in mM) 118 NaCl, 20 TEA, 5.6 KCl, 1.2 MgCl_2_, 2.6 CaCl_2_, 1 glucose and 5 HEPES (pH 7.4 with NaOH). The intracellular solution contained (in mM) 125 Cs-glutamate, 10 CsCl, 10 NaCl, 1 MgCl_2_, 0.05 EGTA, 5 HEPES, 0.1 cAMP, and 3 MgATP (pH 7.15 with CsOH). For some experiments pipette solution was supplemented with β-NADPH and β-NADP^+^ at 100/10 μM; or H_2_O_2_ at 200 µM. The resistances of patch pipettes, pulled from borosilicate glass and coated with sylgard was 4–6 MΩ when filled with pipette solution. Data was normalized to initial cell size and expressed as fF/pF. Mouse β-cells were identified by the absence of voltage-gated Na^+^ current from a holding potential at −70 mV. Data analysis was with FitMaster software (HEKA Electronik, Lambrecht, Germany) and Origin 7.0 and was statistically evaluated with two-tailed, unpaired Student's t-test or one way-ANOVA followed by Scheffe's post-hoc test. All data are expressed as means ± SEM and p < 0.05 was considered significant.

## 4. Conclusions

Insulin exocytosis is a process finely tuned by at least two glucose-dependent pathways: triggering and amplifying. The mechanisms underlying the triggering pathway are well known, whereas the dynamics of the amplifying pathway have still not been revealed. Nevertheless they are strictly interrelated: amplification of β-cell exocytosis is of no consequence until exocytosis is triggered by Ca^2+ ^[3]. Moreover, amplification of insulin release is glucose-dependent and many metabolic signals have been proposed to regulate its occurrence. NADPH is one of the major candidates involved in this process [49]. Our previous work [24,35], and data presented here, demonstrate that the deSUMOylating enzyme SENP1 plays a pivotal role in insulin exocytosis in response to glucose and metabolically-derived signals. One can conclude that NADPH-dependent amplification of β-cell exocytosis is strictly dependent on triggering Ca^2+^ and is mediated by SENP1.

**Figure 6:**
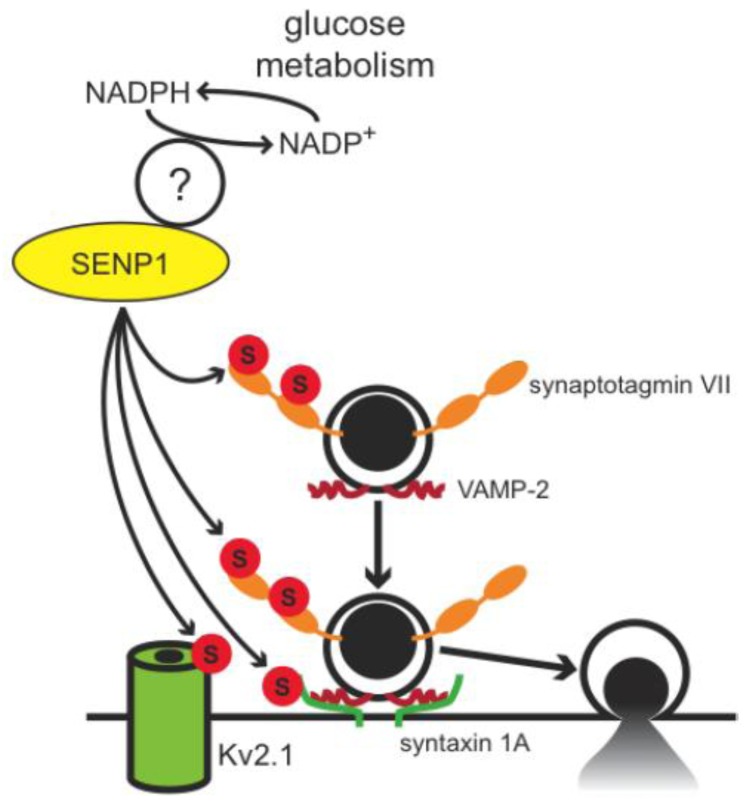
Proposed model for the regulation of insulin exocytosis by deSUMOylation at the exocytotic site. Metabolically derived reducing equivalents, in the form of NADPH, are proposed to amplify insulin exocytosis in part by promoting deSUMOylation of several targets at the exocytotic site. Potential targets include syntaxin 1A, synaptotagmin VII, and the voltage-dependent K^+^channel Kv2.1. Several questions remain, not the least of which include the molecular mechanism linking NADPH to SENP1 activity.

Albeit speculative to indicate which proteins are the SUMO1 targets at the plasma membrane, the exocytotic Ca^2+^-sensor synaptotagmin VII represents a top candidate. Additionally, novel SUMO-targets such as Kv2.1 and syntaxin 1A have been proposed to coordinate insulin exocytosis per se. A proposed model is shown in Figure 6 whereby the metabolic generation of NADPH, perhaps through its role in determining β-cell redox state, acts through SENP1 to mediate the deSUMOylation of a number of targets at the exocytotic site. This removes the ‘brake’ on exocytosis, thus amplifying the secretory response to elevated intracellular Ca^2+^. Of course, much remains to be determined and our understanding of pancreatic β-cell SUMOylation in both the long-term and acute regulation of insulin secretion is in its infancy. Further study of these mechanisms, and the pathophysiological role for SUMOylation, will provide a new layer to our insight into insulin secretion in health and diabetes.
